# Alterations of the Myovesical Plexus of the Human Overactive Detrusor

**DOI:** 10.1155/2014/754596

**Published:** 2014-04-15

**Authors:** Kamiel A. J. Kuijpers, John P. F. A. Heesakkers, Jack A. Schalken

**Affiliations:** Department of Urology, Radboud University Nijmegen Medical Centre, 267 Nijmegen, The Netherlands

## Abstract

*Objectives.* The human bladder shows spontaneous autonomous activity. Detrusor overactivity could be seen as a consequence of exaggerated autonomous activity. Interstitial cells (ICs) play a potential role in coordination of autonomous activity. As it is suggested that changes in ICs coexist with detrusor overactivity (DO), we investigated possible alterations to human bladder ICs. *Methods.* Biopsies were obtained from 23 patients and were categorized into four groups: genuine stress incontinence (without DO) (*n* = 5), neurogenic disease with DO (*n* = 6), bladder outlet obstruction with DO (*n* = 6), or idiopathic DO (*n* = 6). Specimens were processed to investigate expression of N-cadherin and PGP9.5. N-cadherin expression was semiquantitatively analyzed and correlated to PG9.5 expression and bladder wall morphology. *Results.* The population of cells expressing N-cadherin is altered in the overactive detrusor, making no difference between the sources of DO. Punctate distribution of morphological changes was found and downregulation of PGP9.5 expression seemed to coexist with upregulation of N-cadherin expression in the detrusor layer. *Conclusions.* The population of N-cadherin+ cells of the interstitial compartment of the human bladder has the ability to proliferate. As this proliferation seems to coexist with denervation, it could be possible that a highly developed network of interstitial cells replaces the loss of innervation in overactive detrusor.

## 1. Introduction

The overactive bladder (OAB) is a symptomatic diagnosis based on the presence of urgency, with or without incontinence, and is usually accompanied by frequency and nocturia [1]. Its presence imposes a huge burden on the healthcare system, society, and affected individuals [2]. Patients with OAB symptoms and detrusor overactivity (DO) can be divided into three groups; those with neuropathic laesions, those with bladder outlet obstruction (BOO), and those with neither (idiopathic DO) [3].

Although relatively little is known about the aetiology of DO, it is now clear that the human urinary bladder cannot merely be seen as a passive “black box,” solely controlled by neuronal input. Recently, it was found that the isolated bladder shows spontaneous nonneuronal contraction during the filling phase, also known as autonomous activity of the bladder [4]. As frequency and urgency occur during this filling phase, DO can be seen as a consequence of exaggerated autonomous activity during the storage of urine [5].

Two cell types play a potential role in coordination of autonomous activity: interstitial cells (ICs) and intramural neurons of the bladder wall. In the human gastrointestinal tract, specialized ICs of Cajal (ICCs) interconnect through gap junctions and function as pacemakers and conductors of electrical activity between enteric neurons and smooth muscle cells [6]. They hereby coordinate gut peristalsis. ICs are also found in the human bladder, albeit of a different subtypes than the ICs of Cajal [7, 8]. In the bladder, ICs are immunoreactive for the stem cell receptor C-kit, the cytoskeletal filament vimentin, the gap junctional protein connexin-43, the second messenger cyclic guanosine monophosphate (cGMP), and N-cadherin [9–12]. They form a network in the suburothelial area and between the detrusor smooth muscle fascicles. Double labelling confocal microscopy experiments revealed that the ICs are positioned in proximity to nerves [9]. Recent reports have shown that the bladder ICs respond to application of neurotransmitters, firing calcium waves when stimulated by carbachol or ATP [9, 13]. It can therefore be hypothesized that by consecutive interaction of ICs with smooth muscle cells, neuronal firing could consequently result in detrusor muscle activation. Although the exact role for ICs in bladder function has not yet been elucidated, it is highly likely that either quantitative or qualitative changes in bladder ICs coexist with DO. We therefore investigated possible alterations to the network of human bladder ICs in the overactive detrusor using N-cadherin. Additionally, we used PGP9.5 (Protein Gene Product 9.5) as a pan-neuronal marker as it is generally accepted that this protein is expressed by all neuronal structures of the bladder wall [14].

## 2. Methods

### 2.1. Patients

This study was conducted on biopsies from 12 female and 11 male patients, aged 47 to 68 years (mean 60 years), suffering from OAB complaints or genuine stress incontinence. Two cold cup bladder biopsies were obtained from each patient from the posterior bladder wall during cystoscopic procedures. The local ethics committee approved the study and informed consent was obtained from all patients. All patients underwent full urodynamic analysis and were categorized into three groups: neurogenic disease with detrusor overactivity (DO) (*n* = 6; 2 male, 4 female), bladder outlet obstruction (BOO) with DO (*n* = 6; 5 male, 1 female), or idiopathic DO (*n* = 6; 1 male, 5 female). Three females and two males aged 53 to 75 years (mean age 65.6 years) with stress urinary incontinence and urodynamically proven nonoveractive detrusor served as controls. These patients did not suffer from neurogenic disease or bladder outlet obstruction and were all undergoing check cystoscopy. Filling cystometry (50 mL/min) in patients with OAB all revealed DO with a median cystometric bladder capacity (CBC) of 203 mL (range from 28 to 450). The median CMC in control bladders was 400 mL (range from 205 to 500). The CMC of 205 mL was caused by major stress incontinence. In all patients suffering from OAB, intravesical pressure rises during filling were due to DO, as compliance was normal. Acontractile detrusors were not included.

### 2.2. Immunohistochemistry

Bladder specimens were collected and placed in a mould containing Tissue-Tek (Sakura). They were snap-frozen immediately in isopentane at −80°C. Sections of 4 *μ*m were prepared using a cryostat and mounted on Super Frost Plus (Menzel-Gläser) slides. Using haematoxylin-eosin staining techniques, tissue was analyzed for presence of intact urothelium and smooth muscle.

Immunohistochemical staining was performed as previously described by our group [12]. Antibodies against the following markers were used: N-cadherin (rabbit polyclonal antibody); (M142 Takara, mouse monoclonal antibody; C2542 Sigma Clone GC-4), smoothelin [15], and PGP9.5 (mouse monoclonal antibody 7863-0504; AbD Serotec). Antibodies against PGP9.5 were used as it is generally accepted that PGP9.5 is expressed by all neuronal structures of the bladder wall [14, 16, 17]. Negative controls included omission of primary antibodies and incubation with PBS-Extra instead. Positive controls included human prostate cancer specimens [18].

### 2.3. Morphologic Analysis

Immunohistochemical photomicrographs were analyzed semiquantitatively for PGP9.5+ nerve profiles and N-cadherin+ structures. As cold cup biopsies were used, a limited amount of detrusor smooth muscle was available for analysis compared to transmural biopsies. A maximum number of four smooth muscle fascicles per biopsy were photographed. Three slides per specimen were analyzed in duplo. Each set of ten slides was separated by approximately 1 mm of tissue. According to expression of N-cadherin and PGP9.5, each fascicle was semiquantitatively graded as follows: features not present in any photographs, 0; present 0–1/3, +; present in 1/3–2/3, ++; present in 2/3-entire fascicle, +++.

## 3. Results and Discussion

### 3.1. N-Cadherin+ ICs in Control Bladder

All bladder specimens contained the following three layers of tissue: urothelium, suburothelium, and detrusor. Because of the used cold cup biopsy method, the detrusor smooth muscle layer was penetrated to a maximum of two muscle bundles in depth. Full thickness biopsies of human bladder consist of three layers of smooth muscle bundles.

**Figure 1:**
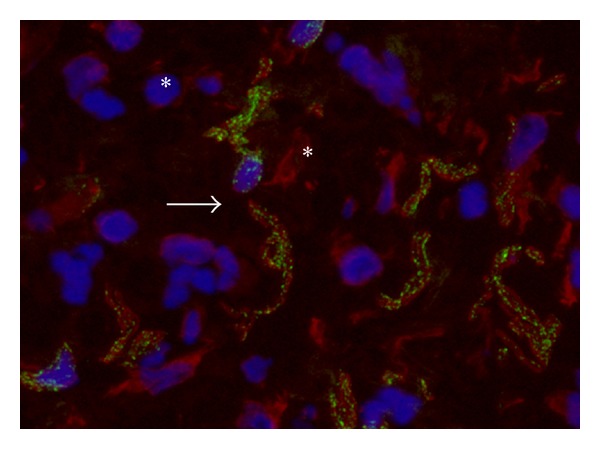
Suburothelial interstitial cells in control bladder. Multiple suburothelial cells with bizarre morphology and multiple processes coexpress a punctate signal for N-cadherin (green) and a filamentous signal for vimentin (red). Nuclei stained with Dapi (blue). Two cells are closely associated with each other (arrow). Cells expressing vimentin but lacking expression of N-cadherin embody fibroblasts (asterisk). Magnification X630. Binocular epifluorescent microscopy.

**Figure 2:**
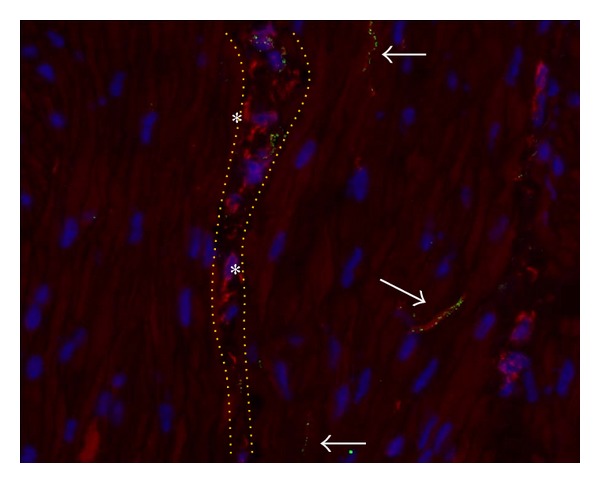
Interstitial cells in the detrusor layer of control bladder. Cells coexpressing N-cadherin (green) and vimentin (red) as found in the detrusor layer. Nuclei stained with Dapi (blue). Note red background signal in smooth muscle cells that was upgraded to facilitate tissue orientation. Arrowheads show slender punctate N-cadherin expression embodying ICs located in the interfascicular clefts (indicated by dotted orange lines). Slender intrafascicular IC-structures (arrows) run in parallel with and between individual smooth muscle cells. Cells expressing vimentin but lacking expression of N-cadherin (asterisk) embody fibroblasts. Magnification X400. Binocular epifluorescent microscopy.

**Figure 3:**
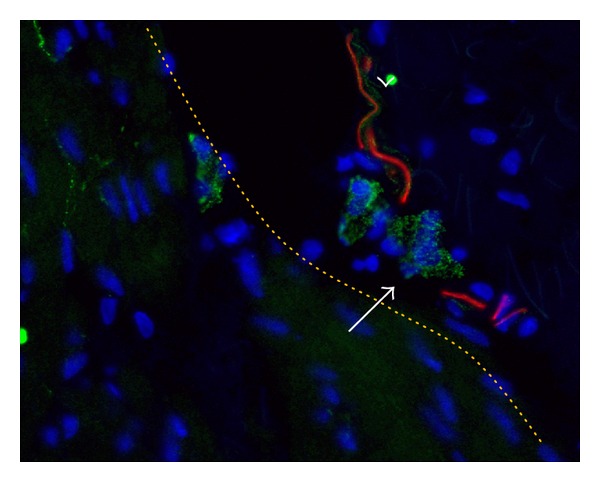
N-Cadherin and PGP9.5. N-Cadherin (green) was double stained with pan-neuronal PGP9.5 (red) and counterstained with Dapi (blue). N-Cadherin positive interstitial cell (arrow) is neighboured by a PGP9.5 positive nerve ending (arrow head). Both cell types do not show coexpression of both markers. Cigar shaped background signal embody collagenous fibres. Enlarged from magnification X400. Binocular epifluorescent microscopy.

According to our previous study [12], N-cadherin positive structures were found throughout the entire bladder wall. They coexpressed vimentin (Figures 1 and 2), but showed no coexpression of the pan-neuronal marker PGP9.5 (Figure 3) or the smooth muscle specific marker smoothelin (data not shown). No immunoreactivity for N-cadherin was found in the urothelial layer (data not shown). N-Cadherin expression profile highly resembled punctate C-kit expression as found by others [16].

N-Cadherin+ cells were located immediately below the urothelium and extended throughout the suburothelial lamina propria into the detrusor layer. They showed a branched morphology with multiple processes that were closely associated (Figure 1). In the detrusor layer, N-cadherin was expressed by cells housed at the border of smooth muscle bundles, perifascicular, and within smooth muscle fascicles (Figure 2).

PGP9.5+ was expressed in all control specimens. PGP9.5+ nerves were often closely associated with N-cadherin+ cells. In the detrusor layer; muscle fascicles were neighboured by primary nerve trunks housed in planes of connective tissue. Smaller nerve branches were found between detrusor fascicles, continuing as small fibers penetrating the fascicles.

### 3.2. Changes to the Population of N-Cadherin+ Cells in the OAB

**Figure 4:**
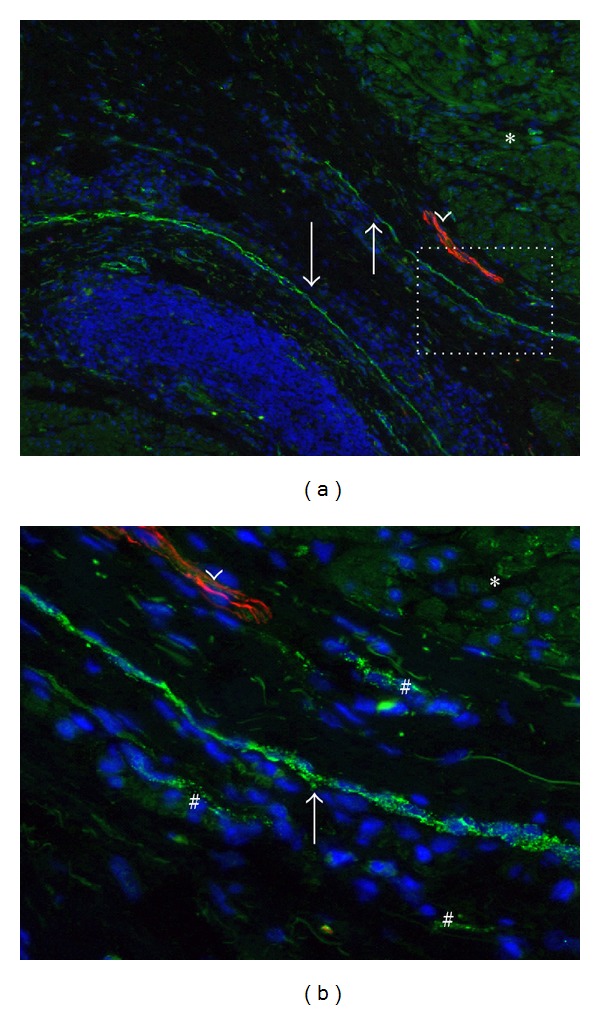
N-Cadherin positive strings at the border of detrusor bundles in overactive detrusor. N-Cadherin (green) double labeled with PGP9.5 (red) and counterstained with Dapi (blue). (a) Two fascicles are almost entirely surrounded by planes of N-cadherin cells (arrows). A large nerve trunk (arrowhead in (a) and (b)) is found in the perifascicular connective tissue. However, note lack of PGP9.5 expression in the upper fascicle. Instead, this fascicle shows a relatively high expression of N-cadherin (asterisk in (a) and (b)). Note green background signal in smooth muscle cells that was upgraded to facilitate tissue orientation. Magnification X100. (b) Magnification of white rectangle in (a). Slender strings consisting of numerous N-cadherin+ cells (arrow) are accompanied by multiple N-cadherin+ cells (#) in their connective tissue coat. Magnification X400. Binocular epifluorescent microscopy.

**Figure 5:**
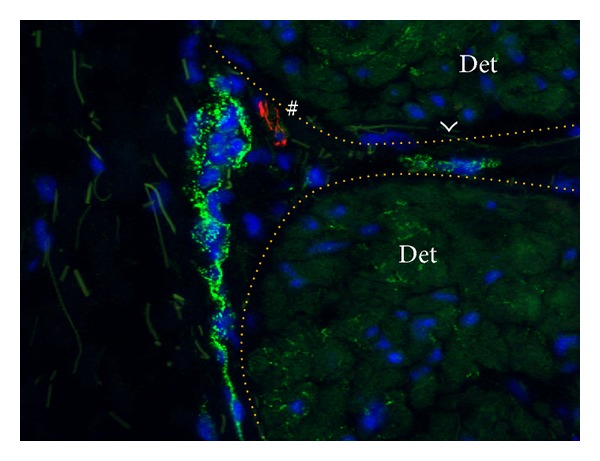
N-Cadherin positive strings and interfascicular cells. N-Cadherin (green) double labeled with PGP9.5 (red) and counterstained with Dapi (blue). Detrusor smooth muscle fascicles (Det) are demarcated by the dotted orange line. Note green background signal in smooth muscle cells that was upgraded to facilitate tissue orientation. N-Cadherin+ string closely associated to an adjacent detrusor smooth muscle fascicle. A cell expressing N-cadherin is housed within the interfascicular connective tissue plane (arrowhead). Note the close association of a PGP9.5+ nerve profile (#) with N-cadherin+ cells. PGP9.5 is not intrafascicularly expressed. Magnification X400. Binocular epifluorescent microscopy.

**Figure 6:**
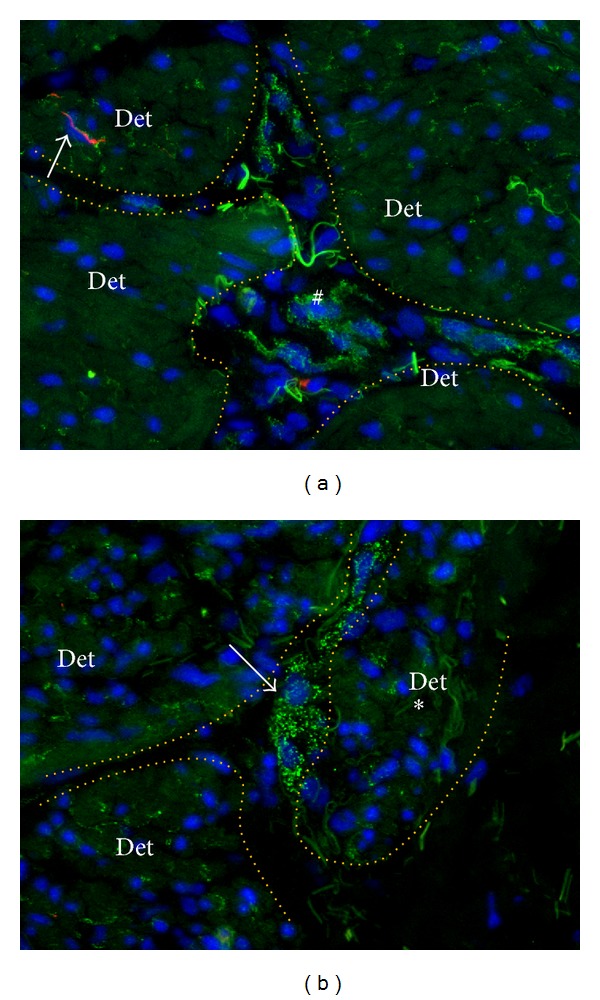
Intrafascicular N-cadherin positive nodes in the overactive detrusor. N-Cadherin (green) double labeled with PGP9.5 (red) and counterstained with Dapi (blue). Note green background signal in smooth muscle cells that was upgraded to facilitate tissue orientation. Detrusor smooth muscle fascicles (Det) are demarcated by the dotted orange line. (a) At the region where interfascicular connective planes meet, N-cadherin+ cells seem to accumulate and form interfascicular nodes (#). Note high level of intrafascicular N-cadherin in combination with low level of PGP9.5 expression (arrow). Filamentous shaped autofluorescent signal embody collagenous fibres. (b) A small group of muscle cells (asterisk) seems to be separated from the main fascicle by a penetrating N-cadherin positive structure (arrow). Magnification X400. Binocular epifluorescent microscopy.

In all specimens, N-cadherin and PGP9.5 expression showed wide variety between smooth muscle fascicles throughout all detrusor muscle layers. In general, N-cadherin expression seemed upregulated in overactive detrusor specimens compared to control specimens, making no difference between the sources of DO (Table 1). In overactive detrusor specimens, changes to the network of N-cadherin+ cells were found at intrafascicular level, perifascicular level, and at the border of smooth muscle bundles. Detrusor smooth muscle bundles with upregulated expression of N-cadherin seemed surrounded by long N-cadherin positive planes (Figure 4). These planes were built up from numerous cells expressing N-cadherin, had a slender morphology, and were housed in a thick layer of connective tissue. At the area in which the interfascicular cleft met the outside of the muscle bundle, the strings were thickened (Figure 5) and seemed to give rise to N-cadherin+ processes running in the interfascicular connective tissue planes. Interfascicular N-cadherin+ processes seemed to junction to nodes (Figure 6). These nodes were not only housed between fascicles, but also between smaller groups of smooth muscle cells. In control specimens, higher expression level of intrafascicular N-cadherin was found in some fascicles as well. However, large strings, interfascicular penetrating structures, and large intrafascicular nodes expressing N-cadherin were never found in control bladders.

The invasion of N-cadherin+ structures did not occur throughout the entire bladder wall but was solely found in smooth muscle bundles that were surrounded by long N-cadherin+ strings. Changes to the IC network were found at each level of depth of the bladder wall and were not restricted to the urothelial side or outer layer of detrusor muscle. However, cold cup biopsies do not enable analysis of full thickness bladder wall morphology. No clear differences between overactive and control specimens were found in the suburothelial N-cadherin+ network.

### 3.3. Smooth Muscle Denervation Coexists with Upgrade of IC Network

**Figure 7:**
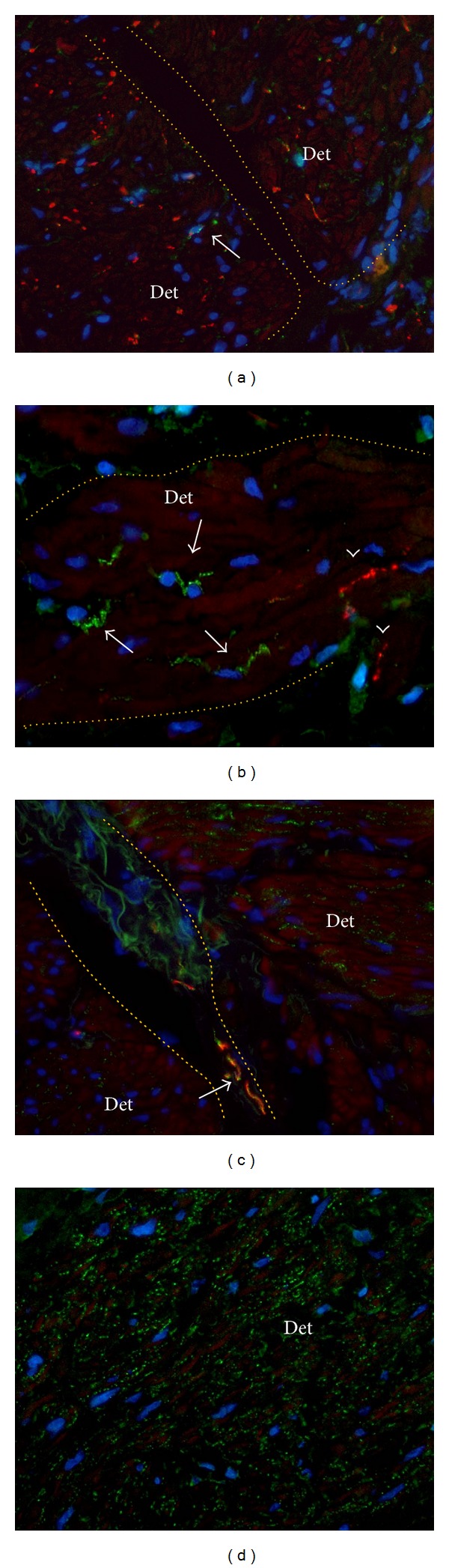
N-Cadherin upregulation and PGP9.5 downregulation in smooth muscle bundles. N-Cadherin (green) was double labeled with PGP9.5 (red) and counterstained with Dapi. Note red background signal in smooth muscle cells that was upgraded to facilitate tissue orientation. Detrusor smooth muscle fascicles (Det) are demarcated by the dotted orange line. (a) Detrusor smooth muscle bundles in normal bladder show a dense PGP9.5 nerve profile in combination with a low level of N-cadherin expression. Arrow shows N-cadherin positive cell. (b) A muscle fascicle in NDO specimen with relatively low PGP9.5 expression (arrowheads) and a relatively high level of N-cadherin+ ICs (arrows). Magnification X630. (c) Cryosection of NDO specimen showing PGP9.5 negative muscle fascicles with high level of punctate N-cadherin expression. Note that PGP9.5+ nerve profiles in the interfascicular cleft closely associate with N-cadherin+ structures (arrow). (d) Smooth muscle fascicle with severely upregulated expression of N-cadherin. Note that the intensity of N-cadherin expressions seems higher than PGP9.5 expression in normal bladder as shown in (a). Magnification X400. Binocular epifluorescent microscopy.

Intrafascicular expression of N-cadherin and PGP9.5 both were heterogeneously expressed and varied from low, to intermediate, and high level of expression (Figure 7). In general, the degree of immunoreactivity for N-cadherin seemed correlated with the level of PGP9.5+ innervation profile. Low expression level of N-cadherin was found to coexist with a high level of PGP9.5+ innervation grade (Table 1). In other fascicles, intermediate level of N-cadherin+ structures coexisted with an intermediate level of PGP9.5 expression. Smooth muscle fascicles with limited or no expression of PGP9.5+ nerve profiles showed intense expression of N-cadherin.

We observed that although PGP9.5+ denervation was found, PGP9.5+ structures were still found in the interfascicular cleft (Figure 7(c)). Large PGP9.5+ nerve trunks located in connective tissue planes outside smooth muscle bundles seemed not downregulated in overactive detrusor specimens.

### 3.4. Discussion

Understanding the pathophysiological mechanism behind detrusor overactivity (DO) is a challenge, as the bladder wall has a complicated structure. While the molecular basis for cell-cell communication between urothelium, neurons, and detrusor smooth muscle cells is getting more and more clear, the exact function of interstitial cells in the human bladder remains unelucidated.

The human urinary bladder shows spontaneous localized and propagating contractions during the storage phase [5]. The bladder may share characteristics with peristaltic activity in the gastrointestinal tract [19, 20]. In the gut, the myenteric plexus controls peristalsis. This plexus is embodied by the intramural neurons and interstitial cells of Cajal (ICCs) [6]. It has recently been proposed that the human bladder, like the gut, consists of modules that contract independently or synchronously with neighbouring modules [21]. Furthermore, the modules would be abnormally active and better coordinated in DO, compared to the normal bladder.

According to this hypothesis, two cell types play a potential role in coordination of autonomous activity in the bladder: interstitial cells (ICs) and intramural neurons. ICs of the bladder share properties of the ICCs [7, 8]. However, they embody a different subtype than the ICCs [7, 8]. In the human bladder, ICs are immunoreactive for the stem cell receptor C-kit, the cytoskeletal filament vimentin, the gap junctional protein connexin-43, the second messenger cyclic guanosine monophosphate (cGMP), and N-cadherin [9–12]. It is highly likely that they form a network in the suburothelial area and between the detrusor smooth muscle fascicles. Although the exact role for ICs in bladder function has not yet been described, it seem that either quantitative or qualitative changes in bladder ICs coexist with increased excitability in the OAB. We therefore investigated possible alterations to the network of human bladder ICs using N-cadherin.

In our study, all biopsies possessed a large population of N-cadherin+ cells. During previous studies we found that N-cadherin most probably can be used as a marker for a subpopulation of bladder ICs [12]. N-Cadherin+ ICs were located in the suburothelial lamina propria and the detrusor layer. In the lamina propria, they showed a bizarre morphology with multiple processes that seemed to form a network. In the detrusor, N-cadherin+ ICs were housed at the border of smooth muscle bundles, perifascicular, and within smooth muscle fascicles. Throughout the bladder wall, ICs expressing N-cadherin were closely associated with PGP9.5 positive neurons. Therefore, these cells could form a myovesical plexus as found in the human gut. Possibly, neuronal information is received in the larger ICs at the border of bundles and passed through the intermediate N-cadherin positive structures to small groups of detrusor smooth muscle cells.

We found changes to the network of N-cadherin+ ICs in most overactive detrusor specimens. These specimens revealed a remarkable variation in N-cadherin expression between and even within detrusor fascicles. This heterogeneous expression profile of N-cadherin is not based on processing artefacts, as in duplo analysis showed exact corresponding pattern of expression.

In the overactive detrusor specimens, upregulation of N-cadherin expression was found at three levels according to anatomical degree, in terms of smooth muscle bundles (level 1), smooth muscle fascicles (level 2), and smooth muscle cells (level 3). It seemed as if large N-cadherin positive structures surrounding smooth muscle bundles were newly formed (level 1), continuing as interfascicular N-cadherin+ processes junctioning to nodes (level 2) and slender intrafascicular N-cadherin+ branches possibly interacting with smooth muscle cells forming small modules.

In fascicles with high expression of N-cadherin, PGP9.5 expression seemed severely downregulated. The intensity of integration of N-cadherin+ structures between smooth muscle cells shows remarkable resemblance with morphological profile of intrafascicular PGP9.5+ innervation in normal bladder, albeit that the N-cadherin+ network seems much more developed. It is generally accepted that PGP9.5 is a nonspecific neuronal marker as it is expressed by all neuronal structures of the human bladder wall [14, 16, 17]. Therefore, it seems that the smooth muscle fascicles lacking PGP9.5 expression are actually denervated. Other investigators also found denervation in overactive detrusor and it is believed to be a general feature of pathological fascicles in the overactive bladder [22–24]. It could therefore be proposed that a highly developed network of ICs replaces the loss of innervation of detrusor smooth muscle fascicles in the overactive detrusor.

Kubota et al. claimed to have found an increased population of suburothelial ICs coexpressing C-kit and vimentin in the BOO-guinea-pig model [25]. However, a network of fibroblasts weakly expressing C-kit and vimentin is also present in the human detrusor layer. These cells do not have IC-like ultrastructure [26]. Therefore, the suburothelial C-kit positive cells as found by Kubota et al. might possibly embody fibroblasts instead of ICs.

De Jongh et al. found differences in the number and distribution of cGMP+ ICs in the bladders of guinea pigs with surgically induced bladder outflow obstruction [17]. Unlike us, they found alterations occurring in the suburothelial area. It is known that suburothelial ICs consist of distinct populations of cells [27]. It might very well be possible that N-cadherin+ ICs embody a subpopulation of ICs as identified by cGMP expression which is not upregulated in the overactive detrusor. Also, as their study was performed in guinea pigs, upregulation of N-cadherin+ ICs in the human bladder could be reserved to the detrusor layer, while the population of suburothelial N-cadherin+ ICs remains unaffected.

Others also found a correlation between upregulation of IC-like cells and exaggerated autonomous activity. Imatinib mesylate (Glivec; a specific C-kit receptor inhibitor) had an inhibitory effect on the overactive detrusor [28]. As C-kit labeling showed significantly more IC-like cells in overactive human detrusor than in normal specimens, it seems likely that this inhibitory effect is due to the upregulation of detrusor ICs. Also, reduction of ICCs is seen in syndromes with reduced autonomous activity of the gut, such as Hirschsprung's disease and functional intestinal obstruction [29].

Normal detrusor smooth muscle cells have poor electrical coupling [30]. Local contractions of groups of smooth muscle cells have been shown to occur in the normal bladder, but these did not lead to intravesical pressure rise [31]. Studies of the overactive detrusor ultrastructure demonstrated the existence of ultraclose abutments and protrusion junctions [32]. It was proposed that these might be the routes of spread of electrical activity in the overactive bladder. However, abnormally wide spread propagation of spontaneous activity could not only result from increased coupling between smooth muscle cells, but also from altered properties of the IC network. If we consider denervated fascicles with an upregulation of N-cadherin expression to be overactive, exceeding the number of overactive fascicles beyond a certain threshold might drive the bladder into behaving overactive. Also, as detrusor smooth muscle bundles house pacemaker cells [9], DO might not need the synchronized nerve-mediated smooth muscle excitation in order to develop.

## 4. Conclusions

It seems that the network of N-cadherin positive ICs in human urinary bladder has the ability to proliferate. As upregulation of N-cadherin+ ICs was found to coexist with denervation, it could be proposed that a developed network of interstitial cells replaces the loss of innervation of detrusor smooth muscle fascicles in overactive detrusor. However, further study is needed to gain more insight into the role of this cellular mechanism and its possible role in exaggerated autonomous activity in the pathological bladder.

## Figures and Tables

**Table 1:** Semiquantitative analysis of intrafascicular N-cadherin and PGP9.5 expression in control and overactive detrusor.

	*n*	Fascicle 1		Fascicle 2		Fascicle 3		Fascicle 4	
		N-cad	PGP9.5	N-cad	PGP9.5	N-cad	PGP9.5	N-cad	PGP9.5
GSI	1	+	++	+	+	+	++	+	++
		+	++	+	++	+	++	+	+++
	2	++	++	+	++	++	−	Error	Error
		+	−	+	++	+	+	+	++
	3	+	++	+	++	+	+	+	+
		+	++	+	+	+	++	+	+++
	4	+	+	++	++	++	−	+	+
		++	++	+	++	+	+	+	++
	5	+	++	+	+	+	++	+	++
		+	++	+	++	+	++	+	+

NDO	1	++	+	++	+	++	+	Error	Error
		+	+	++	++	+++	−	++	−
	2	+	+	++	+	+	+	++	+
		+	−	+	−	++	+	++	−
	3	+	+	++	++	+	+	++	+
		++	+	+	+	+	−	++	−
	4	++	+	Error	Error	+	+	Error	Error
		+	−	++	+	++	−	+++	−
	5	++	+	++	+	+	++	+	++
		+	−	+++	−	+	+	++	+
	6	+	+	Error	Error	++	+	+	+
		+	−	++	+	+	−	+++	−

BDO	1	+	+	++	+	+	+	++	+
		++	+	+	−	+++	−	++	−
	2	+	+	+	+++	+	++	++	+
		++	−	++	+	++	+	++	+
	3	++	+	++	+	++	+	Error	Error
		++	−	+	+	++	−	++	−
	4	+	+++	Error	Error	++	+	+	+
		+++	−	+++	−	++	−	++	−
	5	+	+	+	++	+	+	+	++
		+	++	+++	−	+++	−	+	++
	6	+	+	++	++	+	+	+++	+
		++	+	++	++	+	+++	+	++

IDO	1	++	+	+	+	+++	+	+	+
		+++	−	++	−	+++	−	++	++
	2	Error	Error	++	+	Error	Error	++	+
		++	+	+	+	+++	−	++	−
	3	+	++	+	++	++	+	++	+
		+	−	++	+	+	++	++	−
	4	+++	+	++	+	+++	+	Error	Error
		+	+	+++	−	++	−	++	−
	5	++	+	++	+	Error	Error	+	+
		++	++	++	+	++	−	+	++
	6	++	+	+	+++	+	+++	+++	−
		+	++	+++	−	+++	−	+	++

Smooth muscle fascicles were semiquantitatively analysed for intrafascicular expression of N-cadherin and PGP9.5, and graded as follows: features not present in any photographs, − (no expression); present >0–1/3, + (low expression); present in 1/3–2/3, ++ (intermediate expression); present in 2/3–entire fascicle, +++ (high expression). BDO: DO due to bladder outlet obstruction; NDO: neurogenic DO; IDO: idiopathic DO; GSI: genuin stress incontinence = control bladder. Note the heterogeneity of N-cadherin and PGP9.5 expression profile throughout the control and overactive specimens. In some cases, fatty tissue texture complicated specimen processing. Therefore, we were unable to study some fascicles (see: error).
